# Exploring the Modulation of the Complex Folding Landscape of Human Telomeric DNA by a Low Molecular Weight Ligand

**DOI:** 10.1002/chem.202501377

**Published:** 2025-05-02

**Authors:** Ines Burkhart, Julia Wirmer‐Bartoschek, Janez Plavec, Harald Schwalbe

**Affiliations:** ^1^ Institute for Organic Chemistry and Chemical Biology, Center for Biomolecular Magnetic Resonance (BMRZ) Johann Wolfgang Goethe University Max von Laue Str. 7 60438 Frankfurt am Main Germany; ^2^ Slovenian NMR Centre National Institute of Chemistry Ljubljana SI‐1000 Slovenia

**Keywords:** DNA folding kinetics, DNA ligand interaction, DNA structures, G‐quadruplexes, NMR spectroscopy

## Abstract

Telomeric DNA forms G‐quadruplex (G4) structures. These G4 structures are crucial for genomic stability and therapeutic targeting. Using time‐resolved NMR and CD spectroscopy, we investigated how the ligand Phen‐DC_3_ modulates the folding of the human telomeric repeat 23TAG DNA. The kinetics are modulated by the ligand and by the presence of potassium cations (K^+^). Ligand binding to G4 occurs via a triphasic process with fast and slow phases. Notably, for the G4 structure in the presence of K^+^, the slow rate is ten times slower than without K^+^. These findings offer key insights into the modulation of the complex folding landscape of G4s by ligands, advancing our understanding of G4‐ligand interactions for potential therapeutic applications.

## Graphical abstract

Time‐resolved nuclear magnetic resonance (NMR) and circular dichroism (CD) spectroscopy were utilized to investigate the complex G‐quadruplex folding landscape of the human telomeric DNA in the presence of the ligand Phen‐DC_3_. Our findings revealed significant differences in the kinetics of the pathways originating from the unfolded or K^+^‐bound state.

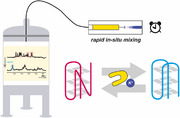

## 1 Introduction

DNA G‐quadruplexes (G4s) are noncanonical DNA structures formed in guanine‐rich sequences, consisting of stacked guanine tetrads stabilized by Hoogsteen hydrogen bonds and the monovalent cations K^+^ or Na^+^.^[^ ^1^, ^2^ ^]^ G4s are distributed across key genomic regions,^[^ ^3^ ^]^ including telomeres,^[^ ^4^, ^5^ ^]^ oncogene promoters,^[^ ^6^, ^7^, ^8^, ^9^ ^]^ and replication origins where their transient formation is involved in transcriptional regulation,^[^ ^10^, ^11^ ^]^ genome stability,^[^ ^12^ ^]^ and replication.^[^ ^13^ ^]^ Consequently, G4s emerged as a drug target for novel therapeutic strategies. DNA G4s exhibit structural polymorphism with diverse topologies influenced by sequence^[^ ^14^, ^15^, ^16^, ^17^ ^]^ and cation^[^ ^18^ ^]^ type due to their complex folding energy landscapes.^[^ ^19^ ^]^ Their strong polymorphism poses challenges for designing suitable ligands, as conformational plasticity in G4s encompasses global topologies whose modulations are difficult to predict at the onset of ligand optimization campaigns. In addition to the structural differences of the polymorphs, their folding kinetics also differ substantially in terms of rates and the population of folding intermediates. Investigating the folding kinetics of G4s whose folding and unfolding and its modulation by low molecular weight ligands is intimately linked to their function.

The 23TAG DNA sequence, derived from human telomeric repeats (TTAGGG), forms G4s and adopts two different hybrid forms in the presence of K^+^ ions.^[^ ^20^, ^21^ ^]^ Widely accepted as a model system, 23TAG is used to study the structural and dynamic properties of telomeric G4s that are crucial for telomere maintenance and cellular aging.^[^ ^5^ ^]^ K^+^‐induced folding of 23TAG has been extensively studied,^[^ ^22^, ^23^, ^24^, ^25^ ^]^ revealing a folding mechanism that follows kinetic partitioning. The final stable state adopts a hybrid conformation, while additional topologies appear even as kinetically favored intermediates.^[^ ^21^ ^]^ Reaching thermodynamic equilibrium is slow due to the transient formation of long‐lived intermediates. By nuclear magnetic resonance spectroscopy (NMR) spectroscopy, Sket et al. identified folding intermediates including an antiparallel chair formed at a pH of 7, and a G‐triplex and hairpin formed at lower pH values.^[^ ^26^ ^]^ These findings have been confirmed by AFM imaging^[^ ^27^ ^]^ and smFRET.^[^ ^28^ ^]^ G4 folding intermediates significantly influence the G4 folding rates.^[^ ^29^ ^]^

**Figure 1:**
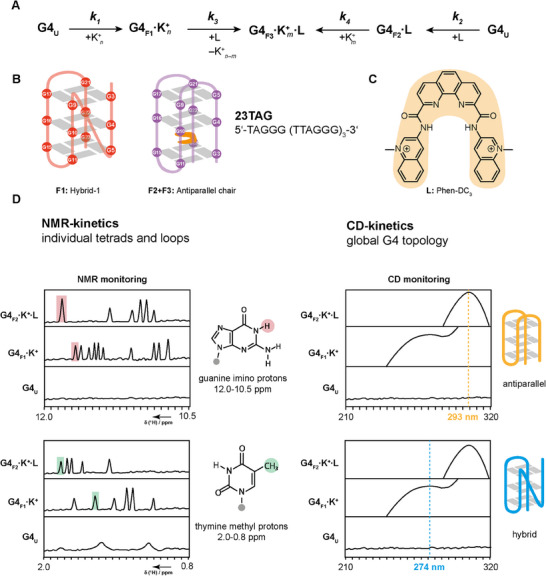
(A) Kinetic scheme assuming sequential folding of K^+^‐bound G4‐DNA (G4F1) and G4:ligand complex (G4_F2_ and G4_F3_) from unfolded DNA (G4_U_). (B) Sequence of human telomeric sequence 23TAG and the two investigated G‐quadruplex topologies in this work. In the presence of K^+^, the major form of 23TAG is hybrid‐1 (red, F1). After the addition of Phen‐DC_3_ (L, structure shown in (C), 23TAG changes the topology to an antiparallel chair (purple, F3), with Phen‐DC3 intercalating between the two lower tetrads. In the absence of K^+^, 23TAG also forms an antiparallel chair (F2) after the addition of Phen‐DC_3_. (D) Spectroscopic techniques illustrated schematically to investigate the kinetics of G4‐ligand folding events. By NMR, guanine imino and thymine methyl protons are traced to obtain insights on individual tetrads and the loop structure, respectively. CD‐spectroscopy allows monitoring of global changes within the G4 topology during the event of ligand binding.

To develop specific G4‐binding ligands as starting points for therapeutic intervention, a vast number of ligands have been synthesized.^[^ ^30^, ^31^ ^]^ Such synthesis programs are supported by structural insights into telomere G4‐ligand complexes from NMR studies.^[^ ^32^, ^33^, ^34^, ^35^, ^36^ ^]^ Typically, the ligand stabilizes the hybrid form by stacking on terminal G‐tetrads. Plavec et al. studied G4 complexes with Phen‐DC_3_.^[^ ^37^ ^]^ Phen‐DC_3_ is a bisquinolinium‐phenanthroline derivative (Figure 1C) with high specificity^[^ ^38^, ^39^ ^]^ and affinity for G4 structures. It affects telomerase activity^[^ ^40^ ^]^ and regulates gene expression,^[^ ^41^ ^]^ making it a key tool compound for studying G4 biology and developing G4‐targeting therapies. However, the kinetics of ligand binding and subsequent G4 DNA conformational switches have remained unexplored. Here, we use both, time‐resolved NMR and CD‐spectroscopies to study the kinetics of ligand‐induced folding of 23TAG and its K^+^‐dependence (Figure 1A). As previously shown, real‐time NMR is a powerful tool to investigate dynamic processes on a late millisecond‐to‐second timescale.^[^ ^14^, ^17^, ^21^, ^42^, ^43^ ^]^

In addition to site‐resolved monitoring of G‐tetrad formation offered by NMR, CD provides information on changes in the overall topology of G4s during folding. 23TAG (dTAGGG(TTAGGG)3) is a natural human telomeric sequence that adopts a hybrid‐1 topology as the thermodynamically major state (Figure 1B, *k_1_*) in the presence of K^+^. It forms a 3+1 G‐tetrad core with the three G‐tetrads stabilized by interactions of deoxynucleotides G9·G3·G21·G17, G10·G16·G22·G4, and G11·G15·G23·G5. In addition to this major state, the 1D ^1^H‐NMR spectrum of 23TAG shows a second minor state with a hybrid‐2 topology.^[^ ^44^ ^]^ Plavec et al. thoroughly investigated the 1:1 G4:ligand complex of 23TAG and Phen‐DC_3_. Their data revealed a ligand binding mode that involves only a single K^+^ between two G‐tetrads. The so‐called “pseudo‐quartet” G3·G11·G15·G23 is stabilized by intercalation of Phen‐DC_3_ between this quartet and the central G‐quartet, inducing a conformational switch from a hybrid to an antiparallel chair topology (Figure 1B).^[^ ^37^ ^]^

## 2 Results and Discussion

Using time‐resolved NMR‐spectroscopy, we here investigated the kinetics of the complex formation in the presence and absence of K^+^ ions. Our data revealed that even in the absence of any monovalent ions, a complex is formed between 23TAG and Phen‐DC_3_, resulting in the same antiparallel chair as with K^+^. The formation of an antiparallel G4 was confirmed by a CD titration of 23TAG with Phen‐DC_3_ in the absence of K^+^. Without K^+^ and Phen‐DC_3_, the CD signal for free ssDNA is observed at 256 nm (Figure S1A). Upon the addition of Phen‐DC_3_, a shift to 292 nm is observed, indicating the formation of an antiparallel G4 structure. This shift is identical to the shift that is observed when Phen‐DC_3_ is added to the K^+^‐bound hybrid G4 formed by 23TAG (Figure S1B) and in agreement with similar experiments by Birkedal et al.^[^ ^45^ ^]^

Rapid injection of K^+^ to this complex of 23TAG and Phen‐DC_3_ (*k_4_*) did not change the imino proton pattern and was not kinetically traceable. In contrast, by in situ injection of ligand solution during the measurement, we were able to extract kinetic rates for both, the decrease of either the unfolded state or the hybrid‐1 state and the build‐up of the antiparallel species similar to the K^+^‐induced folding of the telomeric repeat (Table 1, Figures S2–S4).^[^ ^21^ ^]^ In the case of ligand binding to the unfolded state (*k_2_*) we can separate two different phases. Fast rates of k_2,1 _= 5.4 and 8 min^−1^ are detected for the G4 imino signals and the ligand. We assigned this fast phase to report on ligand binding. The second slow rate k_2,2_ of 0.06–0.09 min^−1^ is also detected for both sets of signals. We assign this second phase to the assembly of the final folded antiparallel G4. The behavior of the guanosines resonating in the region of the imino ^1^H‐NMR spectrum was confirmed by time‐resolved CD measurements. Figure S5 and Table S2 show that all kinetic traces reveal biphasic behavior with fast phases (7.3–5.0 min^−1^) in the same regime as in the NMR experiments. The slow phases are slightly faster than measured by NMR. Thus, global effects of the antiparallel G4 formation seem to be consistent with the kinetics of the imino proton signals regarding the fast folding phase. The slow folding phases are faster on a global scale, suggesting that other folding processes are slightly faster compared to the complete formation of the four Hoogsteen‐type H‐bonds.

**Table 1:** Kinetic rates for the folding of the 23TAG:ligand complexes G4_F2_ and G4_F3_ obtained from bi‐ or tri‐exponential fitting of time‐resolved 1D ^1^H NMR‐spectroscopy data. The rates are reported as fitted value ± standard error estimated from nonlinear regression of one time‐resolved NMR signal trace. k_x,1_, k_x,2_, and k_x,3_ refer to the first, second and third rate, respectively. AP (antiparallel) and H (hybrid) assign the G4 topology. X refers to the folding pathway given in Figure 1A. Corresponding fits can be found in Figures 2, S2–S4, and S7.

Residue	k_x,1_ [min^−1^]	k_x,2_ [min^−1^]	k_x,3_ [min^−1^]
	Ligand Binding	Strand Arrangement	Final Fold
**G4_U_ → G4_F2_·L (*k_2_*)**			
G10, G9, G5, G21, G4, G17, G22 (F3, AP)	5.4 ± 0.5	0.09 ± 0.01	–
Phen‐DC_3_	8 ± 1	0.06 ± 0.01	–
**G4_F1_·K^+^→ G4_F3_·K^+^·L (*k_3_*)**			
G4, G11, G17, G22 (F1, H)	2.1 ± 0.2	0.053 ± 0.008	5.2·10^−3^ ± 0.04·10^−3^
G15, G23 (F1, H)	–	0.05 ± 0.01	4.8·10^−3^ ± 0.05·10^−3^
G5 (F1, H)	–	0.06 ± 0.02	3.6·10^−3^ ± 0.1·10^−3^
T7 (F1, H)	5.5 ± 0.9	0.034 ± 0.003	4.0·10^−3^ ± 0.1·10^−3^
G5, G21, G4, G17 (F2, AP)	6.7 ± 0.8	–	4·10^−3^ ± 0.04·10^−3^
Phen‐DC_3_ (F2, AP)	4.7 ± 0.3	–	6·10^−3^ ± 0.09·10^−3^

**Figure 2:**
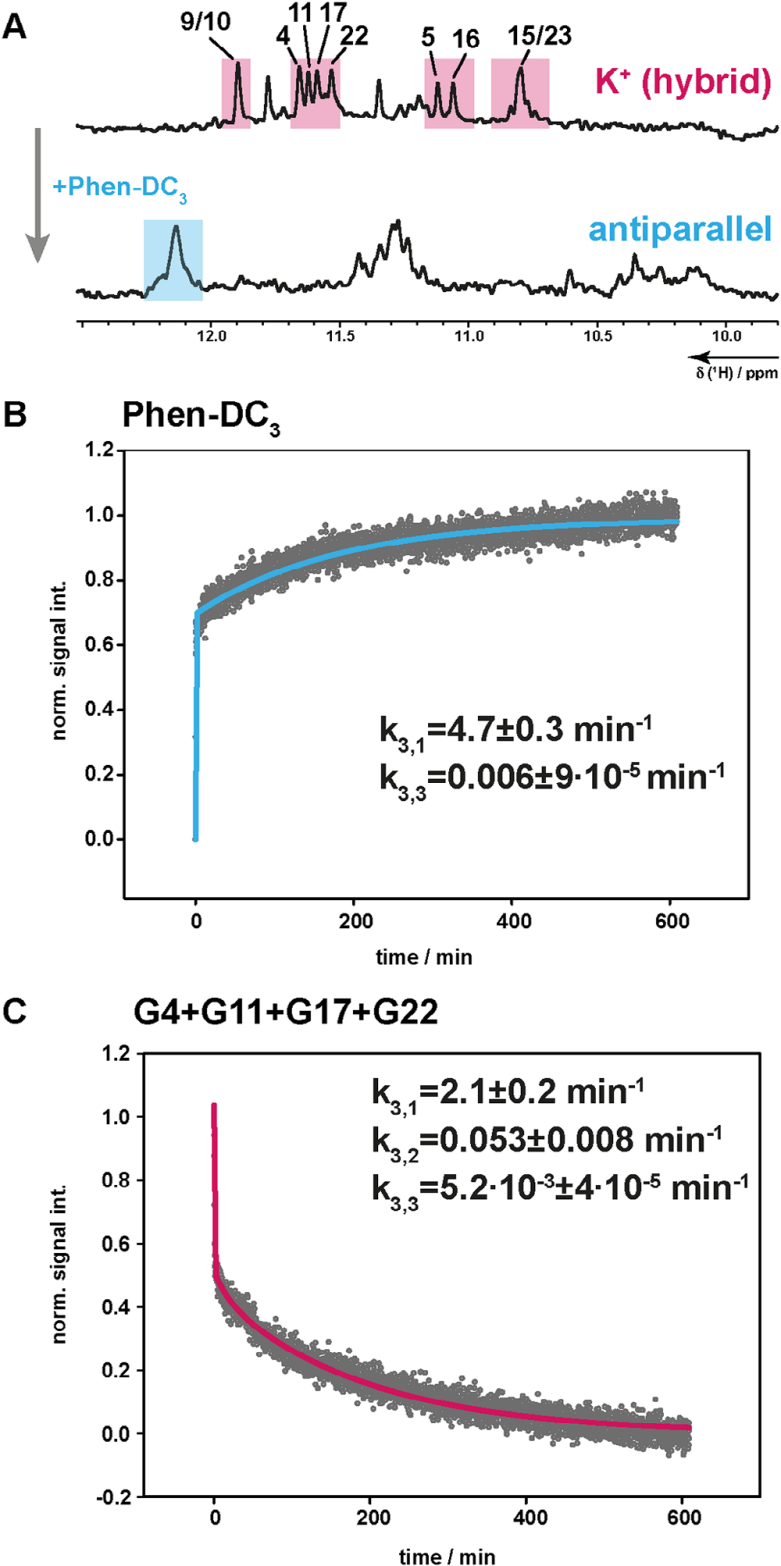
(A) Kinetics of antiparallel G4 formed with 1.5 equivalents of Phen‐DC_3_ in the presence of K^+^. 1D 1H NMR‐spectrum of 23TAG with K^+^ forms a hybrid G4 and refolds to antiparallel upon addition of Phen‐DC_3_ (A). Assignment of the hybrid G4 was transferred from Plavec et al.^[^ 37 ^]^ Kinetic rate constants were taken from the normalized signal integrals from ligand signals and from G4 imino 1H signals, respectively. Data have been fitted with bi‐ or tri‐exponential regression for the Phen‐DC_3_ signal (B, blue) and the G4 signal (C, pink).

For a second set of kinetic investigations, we monitored the rearrangement of an existing, folded G4 state with hybrid topology to another folded G4 state with antiparallel topology (*k_3_*), induced by binding of the ligand and concomitant loss of one potassium ion. Here, we observe up to three phases, depending on the signal‐to‐noise of the associated signals. Two of those three phases are very similar to the phases we observed for folding from the unfolded state (*k_2_*). We thus assign the fastest phase to ligand binding induced changes. A kinetic rate of *k*_3,1 _= 6.7–2.1 min^−1^ is detectable for decaying signals from the hybrid state and upcoming signals from the antiparallel state (Figure 2). The second intermediate rate is associated with the build‐up of signals from states that lack a barrier to fold into the stable antiparallel state, while the third slow rate points to a fraction of G4 states whose refolding from hybrid‐1 to antiparallel G4 state is slower. Thus, the refolding is partitioned into fast‐folding states and slow‐folding states. Using time‐resolved CD spectroscopy (Figure S6), we also observed triphasic kinetics for the decay of the hybrid signal with a fast signal decay of *k*_3,1 _= 8 ± 3 min^−1^.

**Figure 3:**
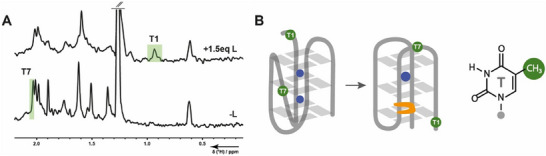
(A) 1D 1H NMR‐spectrum of 23TAG showing the thymine methyl region. Kinetic rate constants were taken from the normalized signal integrals (green). (B) Schematic representation of the structural change of thymidines after the addition of Phen‐DC_3_.

In addition to imino protons, we tracked two different thymidine methyl signals in the ligand‐free (T7) and in the ligand‐bound state (T1) (Figures 3 and S7). The decay of T7 is a triphasic process with fast k_3,1_ and two slower k_3,2_ and k_3,3_. As T7 is located in the middle of the sequence, it is likely that its folding dynamics correspond to the formation of the tetrads and it is more affected by intercalation. T1 on the other hand is a flanking residue that probably has more flexibility and its interactions in the final folded state build up much slower.

## 3 Conclusion

**Figure 4:**
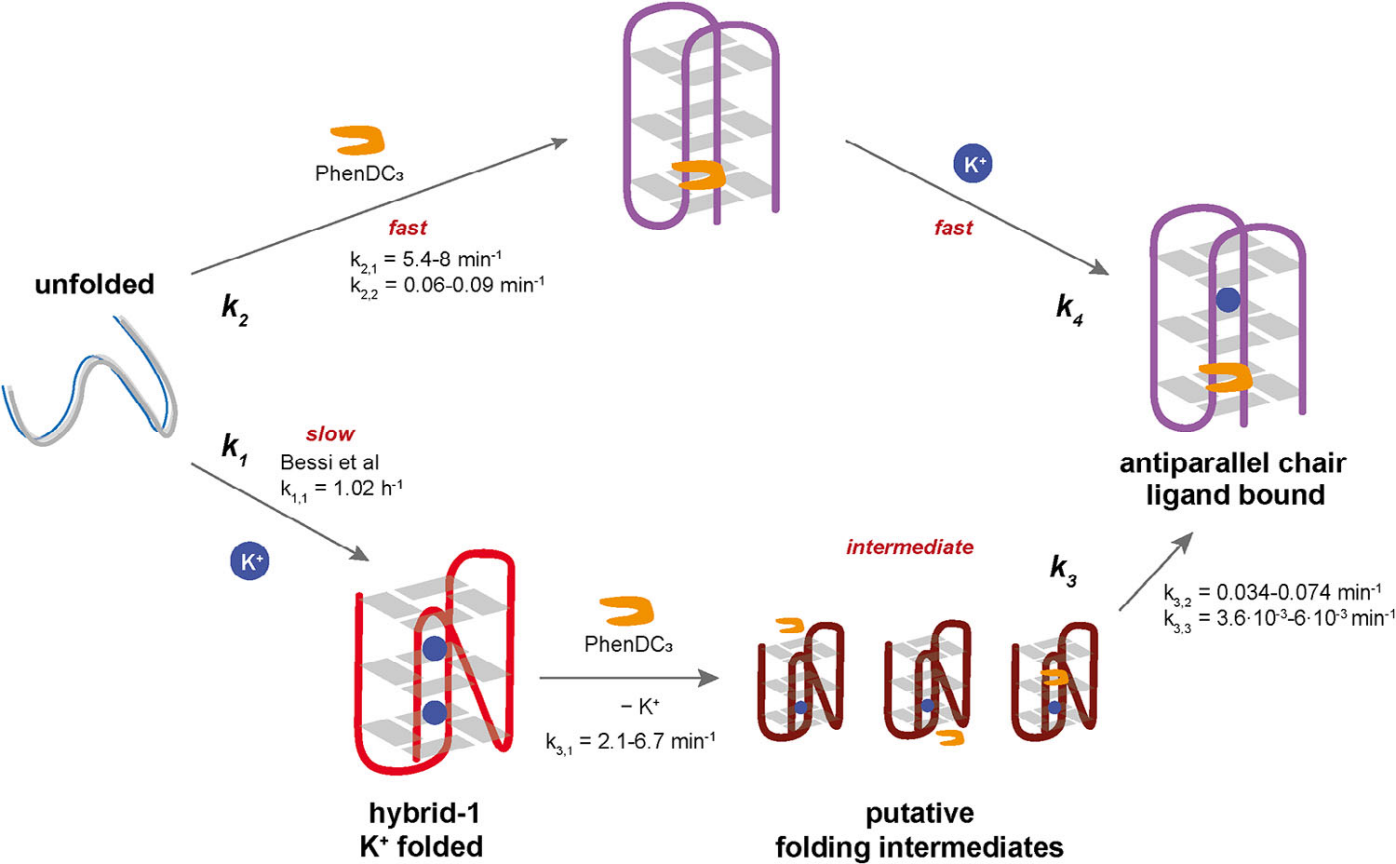
Overview of the investigated folding pathways of the 23TAG‐Phen‐DC_3_ complex. Ligand binding to 23TAG occurs on a faster timescale than K^+^‐induced G4 folding.

In conclusion, our study advances the understanding of folding kinetics and ligand‐induced structural transitions of the 23TAG DNA telomeric G4. Our data reveal a triphasic folding behavior of K^+^‐bound 23TAG in the presence of Phen‐DC_3_, emphasizing the distinct fast and slow kinetic phases influenced by K^+^ ions. The initial, fast phase corresponds to ligand binding, while the second and third slow phases are assigned to strand arrangement and final folding of the antiparallel structure. Unprecedentedly, we explored the folding landscape of a G4 and how it is influenced by ligand binding and demonstrated that the folding kinetics of the ligand:G4 complex are drastically accelerated compared to the K^+^‐bound G4. The process of ligand binding with K^+^ involves the presence of intermediates that impede the overall rate of folding (Figure 4). Likely, these off‐pathway folding intermediates consist of hybrid folds that already have a preformed ligand binding pocket and an ejected K^+^ ion. These need refolding to be able to fold into an antiparallel G4, whereas the ligand‐bound, cation‐free antiparallel state is reached through fast‐folding intermediates. The ability of Phen‐DC_3_ to form a stable antiparallel chair conformation in the absence of K^+^ underlines its potential as a robust G4 stabilizer. Insights into the kinetic behavior of G4s contribute to the development of novel ligands tailored to specific G4s for therapeutic applications, especially, because the accessibility of G4 structures and advances in our understanding of their folding and unfolding are fundamentally linked to biological processes of replication and transcription.

## Supporting Information

The authors have cited additional references within the Supporting Information.^[^ ^46^, ^47^ ^]^

## Conflict of Interests

The authors declare no conflict of interest.

## Supporting information



Supporting information
