# Supplementary material for: Exploring the Modulation of the Complex Folding Landscape of Human Telomeric DNA by a Low Molecular Weight Ligand
Source: Chemistry. 2025 May 2;31(32):e202501377. doi: 10.1002/chem.202501377 (PMC12144896; doi:10.1002/chem.202501377)
Supplement: Supplementary file 1 — Supporting information [file CHEM-31-e202501377-s001.pdf]

Exploring the Modulation of the Complex Folding Landscape of Human Telomeric DNA by a Low Molecular Weight Ligand

Ines Burkhart[1], Julia Wirmer-Bartoschek[1], Janez Plavec[2], Harald Schwalbe[1,*]

1 Institute for Organic Chemistry and Chemical Biology, Center for Biomolecular Magnetic Resonance (BMRZ), Goethe University Frankfurt am Main, 60438 Frankfurt/Main, Hessen, Germany.

2 Slovenian NMR Centre, National Institute of Chemistry, SI-1000 Ljubljana, Slovenia.

Supporting Information

Experimental part

Sample preparation. The oligonucleotide 23TAG (5′-TAGGG $(TTAGGG)_3$-3′) was purchased from Eurofins MWG Operon (Ebersberg, Germany) in HPLC grade and desalted via ultracentrifuge filtration device (2 kDa cut-off). The oligomer was dissolved in ddH_2O and stored at -20 °C. For measurements in the presence of K^+, KPi buffer (20 mM) supplemented with 70 mM KCl and for K^+-free measurements, 20 mM Bis-Tris buffer was used. All buffers were prepared at a pH of 7.0. G4 samples were incubated at 95 °C for 5 min and subsequently folded by slow cooling within 24 h to room temperature.

Time-resolved NMR experiments. NMR spectra were recorded on a Bruker 700 MHz and 800 MHz spectrometer equipped with a cryogenic probe at 298 K. All samples were prepared with 150 µM DNA, 0.1 mM 3-(trimethylsilyl)-1-propanesulforic acid (DSS) as a reference and 10% D_2O at a pH of 7.0. For K^+-free samples, 20 mM BisTris were used, K^+ induced samples were prepared with 20 mM KPi buffer and 70 mM KCl. Ligand solutions were prepared in the same buffers, respectively. Time-resolved NMR experiments were recorded as pseudo-2D experiments[1] using a jump-return-echo pulse scheme to suppress water signals. 16 spectra before and 4080 spectra after injection were recorded with 8.96 s per 1D-spectrum. All NMR spectra have been analyzed by Bruker Biospin software TopSpin 4.0.9. Extracted kinetic traces were fitted with a bi- or tri-exponential function:

$$f(x)=a\cdot(1-e^{(-b\cdot x)})+c\cdot(1-e^{(-d\cdot x)})$$

$$f(x)=a\cdot(1-e^{(-b\cdot x)})+c\cdot(1-e^{(-d\cdot x)})+g\cdot(1-e^{(-h\cdot x)})$$

Time-resolved NMR set-up. Using a special rapid mixing device originally developed by Mok et al., DNA and ligand solutions were mixed inside the NMR spectrometer. A detailed description of the set-up is provided here.[2] Real-time NMR was conducted after rapid mixing of DNA with the ligand via a coaxial insert with a glass capillary tubing and a glass micropipette within the NMR tube. Prior to measurement, the ligand was drawn up into the micropipette together with a small air bubble to prevent leakage. The mixing was performed in a Shigemi tube with 320 µL DNA sample and 40 µL ligand solution. Injection was triggered by a pneumatic injector that was placed outside the magnet and connected to the console of the spectrometer.

Time-resolved CD experiments. CD data were recorded on a Jasco J-810 spectropolarimeter in a 2 mm quartz glass cuvette at 298 K using 7.5 µM DNA for each measurement. For *in situ* mixing, the ligand was injected during the measurement manually through a syringe as part of a homebuilt mixing set-up. Kinetic traces were obtained at single wavelengths of interest and monitored over time. The resulting data were fitted with a bi-or tri-exponential function as described above.

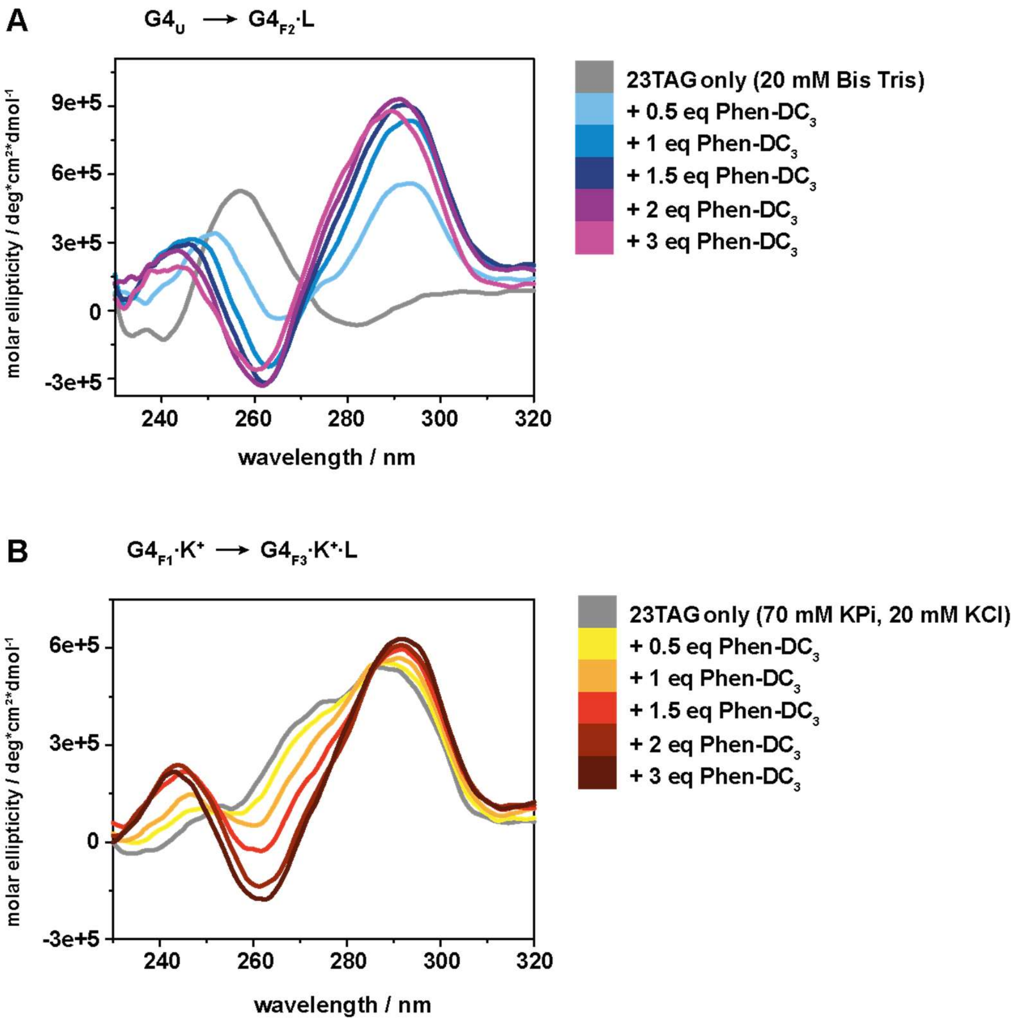

Fig. S1. A) CD titration of 23TAG in the absence of K^+ (20 mM BisTris) with 0.5, 1, 1.5, 2 and 3 equivalents of Phen-DC_3. The ssDNA without K^+ and ligand exhibits a maximum at 256 nm, which undergoes a shift upon the addition of Phen-DC_3 to 292 nm. This shift is evident of the formation of an antiparallel G4. B) CD titration of hybrid-1 23TAG with 0.5, 1, 1.5, 2 and 3 equivalents of Phen-DC_3. A shift from the hybrid to the antiparallel G4 fold is observed.

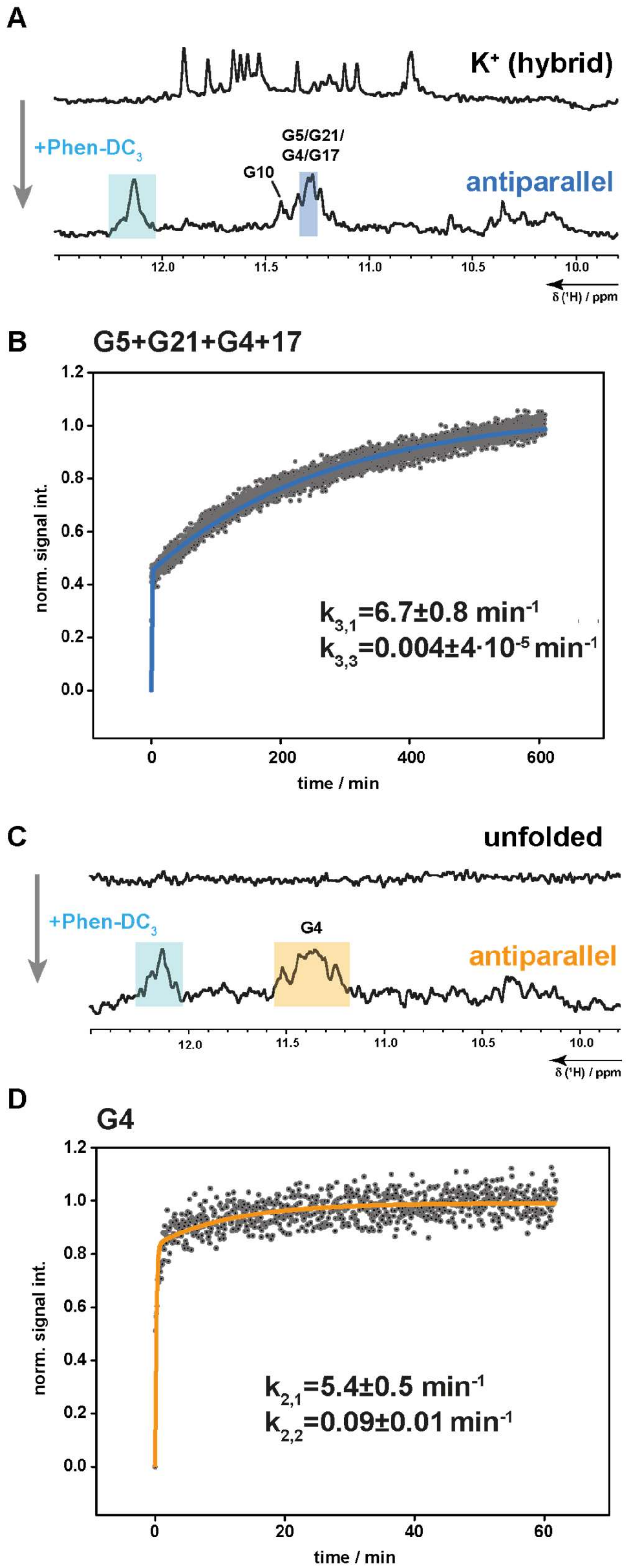

Fig. S2. A) 1D ^{1}H NMR spectrum of 23TAG with K^+ forms a hybrid G4 and refolds to antiparallel upon addition of Phen-DC_3. B) Kinetics of antiparallel G4 formed with 1.5 eq of Phen-DC_3 in the presence of K^+. Kinetic rate constants were taken from the normalized signal integrals in the imino ^{1}H region. Data has been fitted with bi-exponential regression for G4 signal (B, orange) and Phen-DC_3 signal (blue).

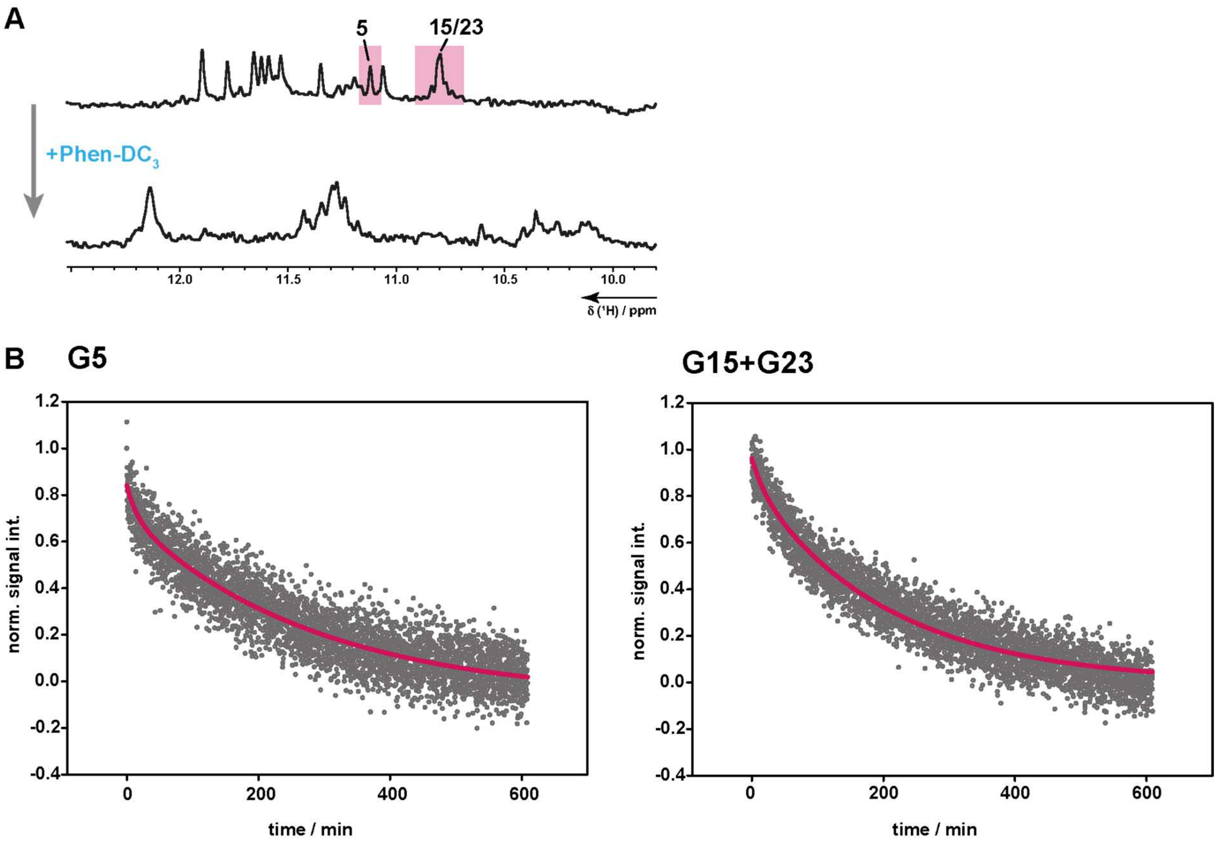

Fig. S3. Kinetics of hybrid G4 signal that decreases upon addition of 1.5 eq Phen-DC_3 in the presence of K^+. 1D ^1H NMR spectrum of 23TAG with K^+ forms a hybrid G4 and refolds to antiparallel upon addition of Phen-DC_3 (A). Kinetic rate constants were taken from the normalized signal integrals in the imino ^1H region. Data has been fitted with bi-exponential regression for the G4 signals (B, pink).

Table S1. Kinetic rate constants for the guanine residues of the hybrid G4 signal.

**Guanosine**	**$k_{3,2}$ / min^{-1}**	**$k_{3,3}$ / min^{-1}**
5	0.06±0.02	$3.6\cdot10^{-3}\pm0.1\cdot10^{-3}$
15+23	0.05±0.01	$4.8\cdot10^{-3}\pm0.05\cdot10^{-3}$

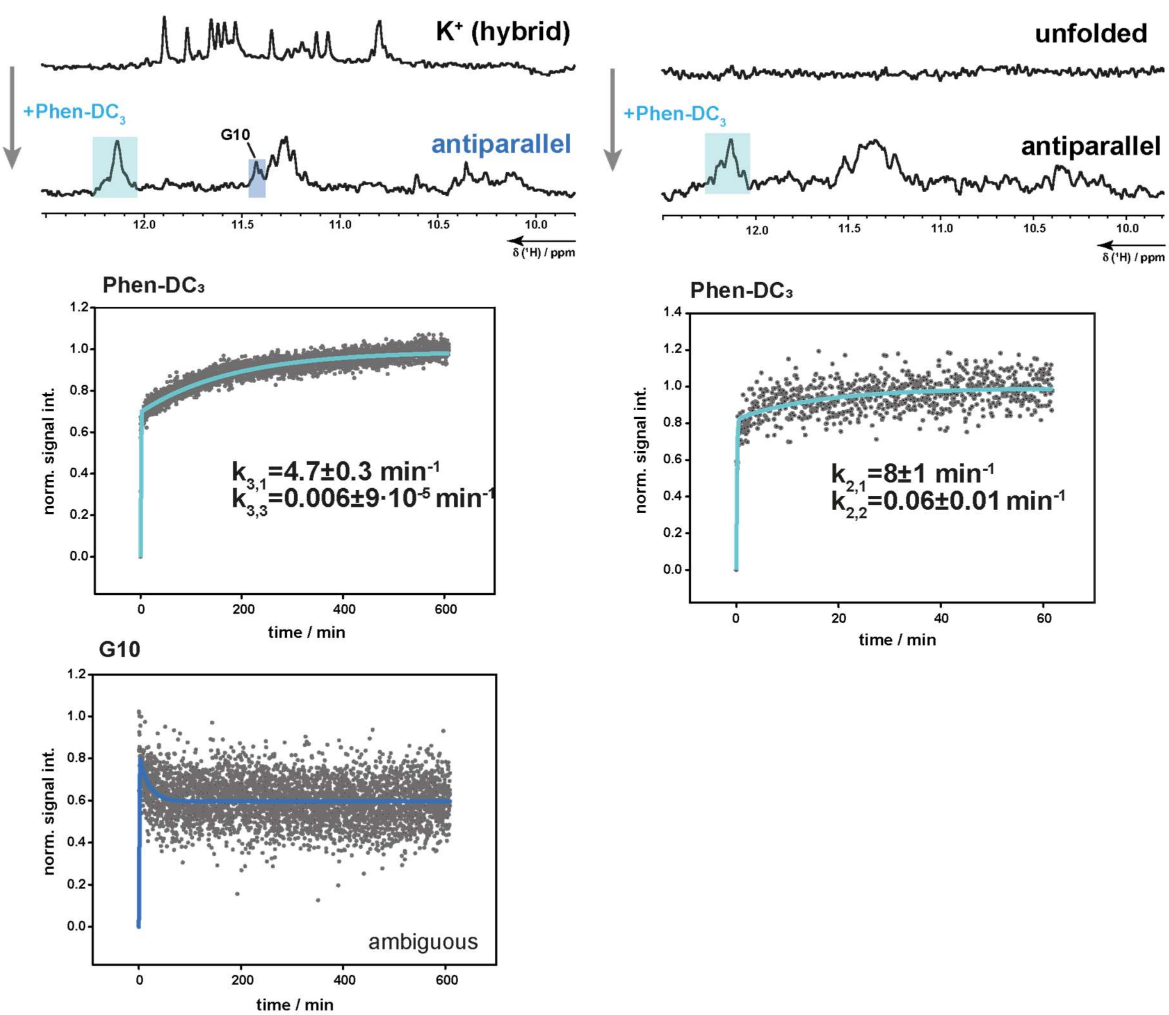

Fig. S4. Kinetics of antiparallel G4 formed with 1.5 eq of Phen-DC3. Kinetic rate constants were taken from the normalized signal integrals in the imino ^{1}H region. Data has been fitted with bi-exponential regression for G4 signal (B, blue) and Phen-DC3 signal (turquoise). Fitting of G10 was ambiguous. Imino ^{1}H region of hybrid and antiparallel 23TAG G4 before and after ligand injection (A).

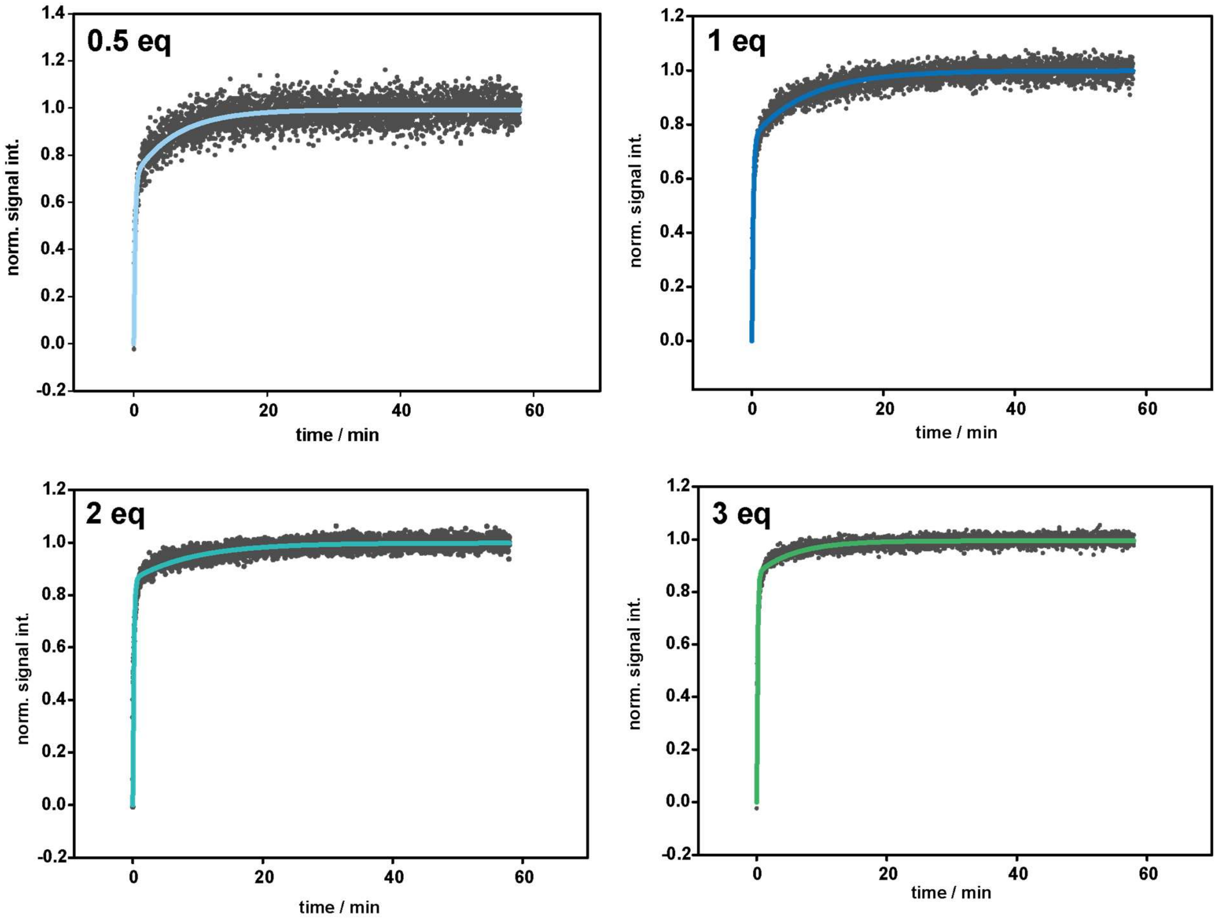

Fig. S5. Kinetics of antiparallel G4 formed with 0.5, 1, 2 and 3 eq Phen-DC_3 in the absence of K^+ by CD-spectroscopy. Kinetic rate constants were taken from the normalized signal integrals. Data recorded at 293 nm to track antiparallel forms has been fitted with bi-exponential regression (A, blue/green lines).

Table S2. Kinetic rate constants for the increase of antiparallel species measured at 293 nm for 0.5, 1, 2 and 3 equivalents of Phen-DC_3 in the absence of K^+.

**Equivalents of Phen-DC_3**	**$k_{2,1}$ / min^{-1}**	**$k_{2,2}$ / min^{-1}**
0.5	6±1	0.16±0.01
1	5.0±0.1	0.119±0.003
2	6.2±0.1	0.859±0.002
3	7.3±0.1	0.171±0.005

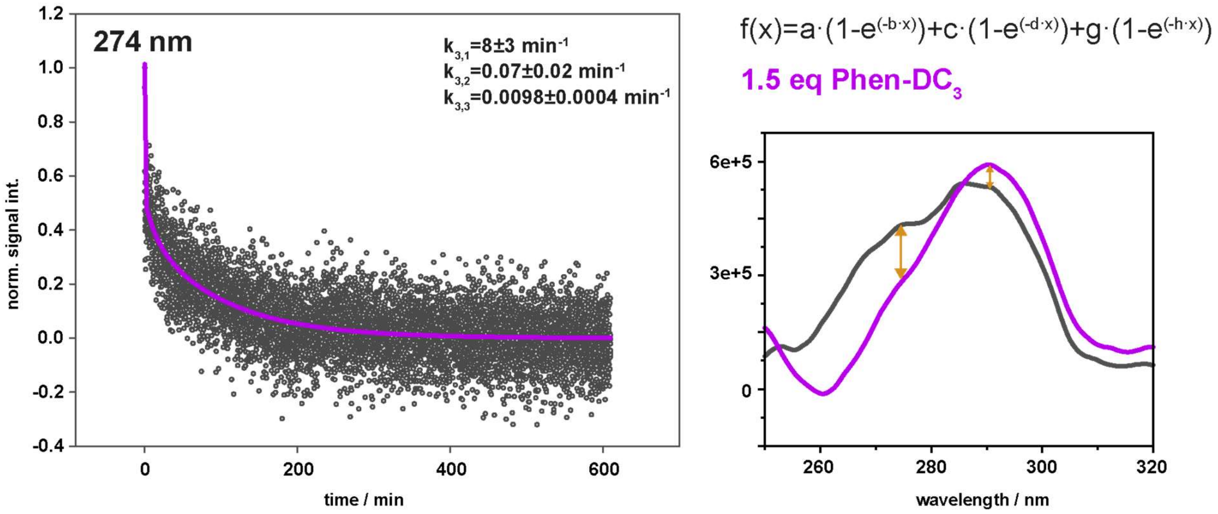

Fig. S6. Kinetics of antiparallel G4 formed with 1.5 eq Phen-DC_3 in the presence of K^+ by CD-spectroscopy. Kinetic rate constants were taken from the normalized signal integrals. Data recorded at 274 nm to track hybrid forms has been fitted with tri-exponential regression (A, purple line).

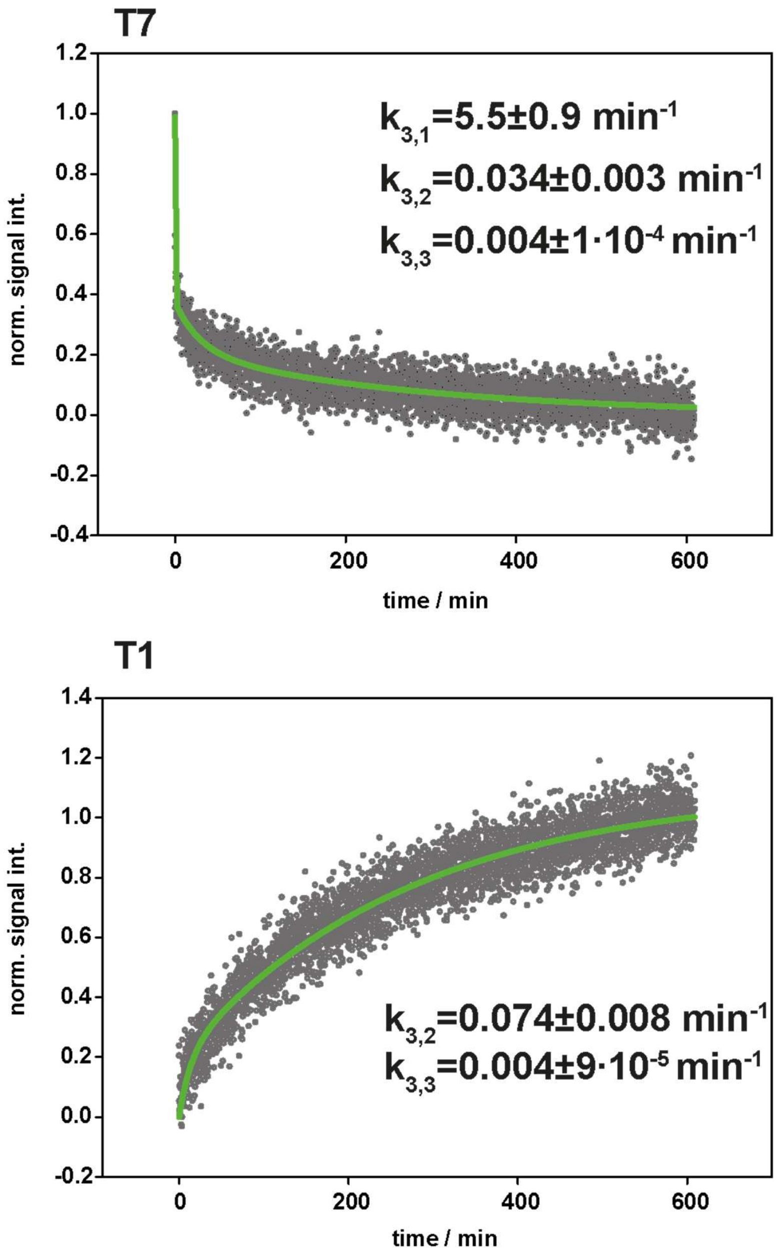

Fig. S7. Kinetics of thymines T1 and T7 of 23TAG from time-resolved NMR data. Kinetic rate constants were taken from the normalized signal integrals. Kinetics of thymines T1 and T7 of 23TAG from time-resolved NMR data. Data has been fitted with bi- or tri-exponential regression. Upon addition of Phen-DC_3, T7 methyl group signal of the hybrid G4 decays and T1 rises due to the structural change.

References

[1] J. Buck, B. Fürtig, J. Noeske, J. Wöhnert, H. Schwalbe, Time-resolved NMR methods resolving ligand-induced RNA folding at atomic resolution, *Proc. Natl. Acad. Sci.* **2007**, *104*, 15699–15704.

[2] K. H. Mok, T. Nagashima, I. J. Day, J. A. Jones, C. J. V. Jones, C. M. Dobson, P. J. Hore, Rapid Sample-Mixing Technique for Transient NMR and Photo-CIDNP Spectroscopy: Applications to Real-Time Protein Folding, *J. Am. Chem. Soc.* **2003**, *125*, 12484–12492.
